# Identification of a Novel Insertion Site HVT-005/006 for the Generation of Recombinant Turkey Herpesvirus Vector

**DOI:** 10.3389/fmicb.2022.886873

**Published:** 2022-05-25

**Authors:** Xusheng Zai, Bin Shi, Hongxia Shao, Kun Qian, Jianqiang Ye, Yongxiu Yao, Venugopal Nair, Aijian Qin

**Affiliations:** ^1^Ministry of Education Key Lab for Avian Preventive Medicine, Yangzhou University, Yangzhou, China; ^2^Jiangsu Co-innovation Center for Prevention and Control of Important Animal Infectious Diseases and Zoonoses, Yangzhou, China; ^3^Joint International Research Laboratory of Agriculture and Agri-Product Safety, The Ministry of Education of China, Yangzhou, China; ^4^The Pirbright Institute & UK-China Centre of Excellence for Research on Avian Diseases, Guildford, United Kingdom

**Keywords:** HVT vectored vaccine, CRISPR/Cas9, insertion site, H9N2, hemagglutinin, influenza virus

## Abstract

Turkey herpesvirus (HVT) has been widely used as a successful live virus vaccine against Marek's disease (MD) in chickens for more than five decades. Increasingly, HVT is also used as a highly effective recombinant vaccine vector against multiple avian pathogens. Conventional recombination, or recombineering, techniques that involve the cloning of viral genomes and, more recently, gene editing methods have been used for the generation of recombinant HVT-based vaccines. In this study, we used NHEJ-dependent CRISPR/Cas9-based approaches to insert the mCherry cassette for the screening of the HVT genome and identifying new potential sites for the insertion of foreign genes. A novel intergenic site HVT-005/006 in the unique long (UL) region of the HVT genome was identified, and mCherry was found to be stably expressed when inserted at this site. To confirm whether this site was suitable for the insertion of other exogenous genes, haemagglutinin (HA) of the H9N2 virus was inserted into this site, and a recombinant HVT-005/006-HA was rescued. The recombinant HVT-HA can grow well and express HA protein stably, which demonstrated that HVT-005/006 is a promising site for the insertion of foreign genes.

## Introduction

Vaccination and biosecurity are two effective strategies for the control of diseases in the poultry industry, which also play an important role in the prevention of infectious diseases. Mass vaccination, particularly in poultry farms where birds are in a relatively confined space, is a rational alternative that has been proven to reduce economic losses from diseases (Atkins et al., 2013). The majority of these vaccines used in the poultry industry are conventional vaccines generated by attenuated or inactivated pathogens, and many of these vaccines have certain disadvantages, such as the increased virulence of the wild-type viruses (Garcia, 2017), induction of adverse reactions in the vaccinated animals (Zhao et al., 2013), interference with the immunity in the presence of maternal antibodies (Hsieh et al., 2010), and the inability to differentiate between infected and vaccinated animals (Dimitrov et al., 2017). To overcome these problems, it is necessary to develop new vaccines for protecting domestic poultry, and the viral vector vaccines are a promising choice in this context.

Vectored vaccines are based on the use of a living, non-pathogenic microorganism to express one or more heterologous antigens in the host. Three live viral vectors commonly used in poultry vaccination are turkey herpesvirus (HVT), fowl pox virus (HVT), and Newcastle disease virus (NDV). While all the three viral vectored vaccines are used as part of the mass vaccination strategy for controlling avian diseases, the effect of maternally derived antibodies (MDAs) is an important factor when considering the choice of these three live viral vectors. MDAs can interfere with the efficacy of live FPV-vectored vaccines (Richard-Mazet et al., 2014) and affect the development of protective immunity with NDV-based vaccines (Bertran et al., 2018). Unlike FPV- and NDV-based vaccines that are not fully effective in the presence of maternal antibodies, HVT has shown good efficacy in the presence of maternal antibodies and the capability of providing life-long immunity (Calnek, 2001).

As a widely used vaccine vector, HVT has been applied to control several avian diseases, including Newcastle disease (ND), avian influenza (AI), infectious bursal disease (IBD), and infectious laryngotracheitis (ILT) by encoding heterologous antigen proteins as dual vaccines (Reddy et al., 1996; Tsukamoto et al., 2002; Li et al., 2011; Vagnozzi et al., 2012). Previously, commercial HVT-vectored vaccines used in the poultry industry usually carried the gene encoding a single foreign antigen. With the development of technology for generating recombinant HVT vaccines, commercial HVT-vectored vaccines with double foreign gene inserts have been licensed in recent years, and a triple insert live avian herpesvirus-vectored vaccine was constructed as a potential vaccine candidate for the protection of poultry against multiple avian viral pathogens in 2020 (Tang et al., 2020). While the generation of HVT-vectored vaccines that carry multiple foreign genes is attractive, this would need the use of suitable insertion sites in the viral vector for stable expression of the foreign gene. Restricted by the limited number of insertion sites found in the HVT genome and the differences in the expression levels of the foreign genes in different insertion sites (Zhang et al., 2014), it is valuable to search for new potential insertion sites to optimize the HVT-vectored vaccine expressing three or more foreign genes. Recombinant HVT vaccines were generated by several methods, such as conventional homologous recombination, cosmid system, bacterial artificial chromosome (BAC), and CRISPR/Cas9 system (Baigent et al., 2006; Liu et al., 2016; Tang et al., 2018). Among these, CRISPR/Cas9 system, the technology that has developed rapidly in recent years, has been applied to many viral vectors, including avian herpesviruses. For the generation of recombinant HVT vaccines by the CRISPR/Cas9 method, there are two pathways: homology-directed repair (HDR) and non-homologous end joining (NHEJ). In this study, NHEJ-dependent CRISPR/Cas9 editing was used to screen the insertion sites and generate HVT recombinants.

In this study, we wanted to screen the HVT genome for new insertion sites for generating recombinant HVT based on NHEJ-dependent CRISPR/Cas9. We report the identification of HVT-005/006 as a potential insertion site that was used for the expression of the mCherry reporter cassette. Subsequently, the HA gene of the H9N2 virus was successfully inserted into this locus, and the recombinant HVT-005/006-HA was shown to be stable after 15 passages, which displayed good expression with no adverse effects on the growth of the recombinant virus. Our results showed that the HVT-005/006-HA could be examined as a candidate vaccine for providing protection against the H9N2 influenza virus.

## Materials and Methods

### Viruses and Cell Culture

The HVT Fc126 vaccine strain (Okazaki et al., 1970) was provided by Qianyuanhao Biological Co., Ltd. Primary chick embryo fibroblasts (CEFs) were prepared from 9-day-old embryos, grown in Dulbecco's Modified Eagle's Medium (DMEM) (Thermo Fisher Scientific, Shanghai, China) supplemented with 5% fetal bovine serum (Thermo Fisher Scientific, Shanghai, China), and maintained in DMEM with 1% fetal bovine serum, 10% tryptone phosphate broth (Sigma, Shanghai, China), and 100 units/ml of penicillin and streptomycin (Thermo Fisher Scientific, Shanghai, China) at 37°C in 5% CO_2_ atmosphere.

### Construction of the sgRNA and Donor Plasmids

**Figure 1:**
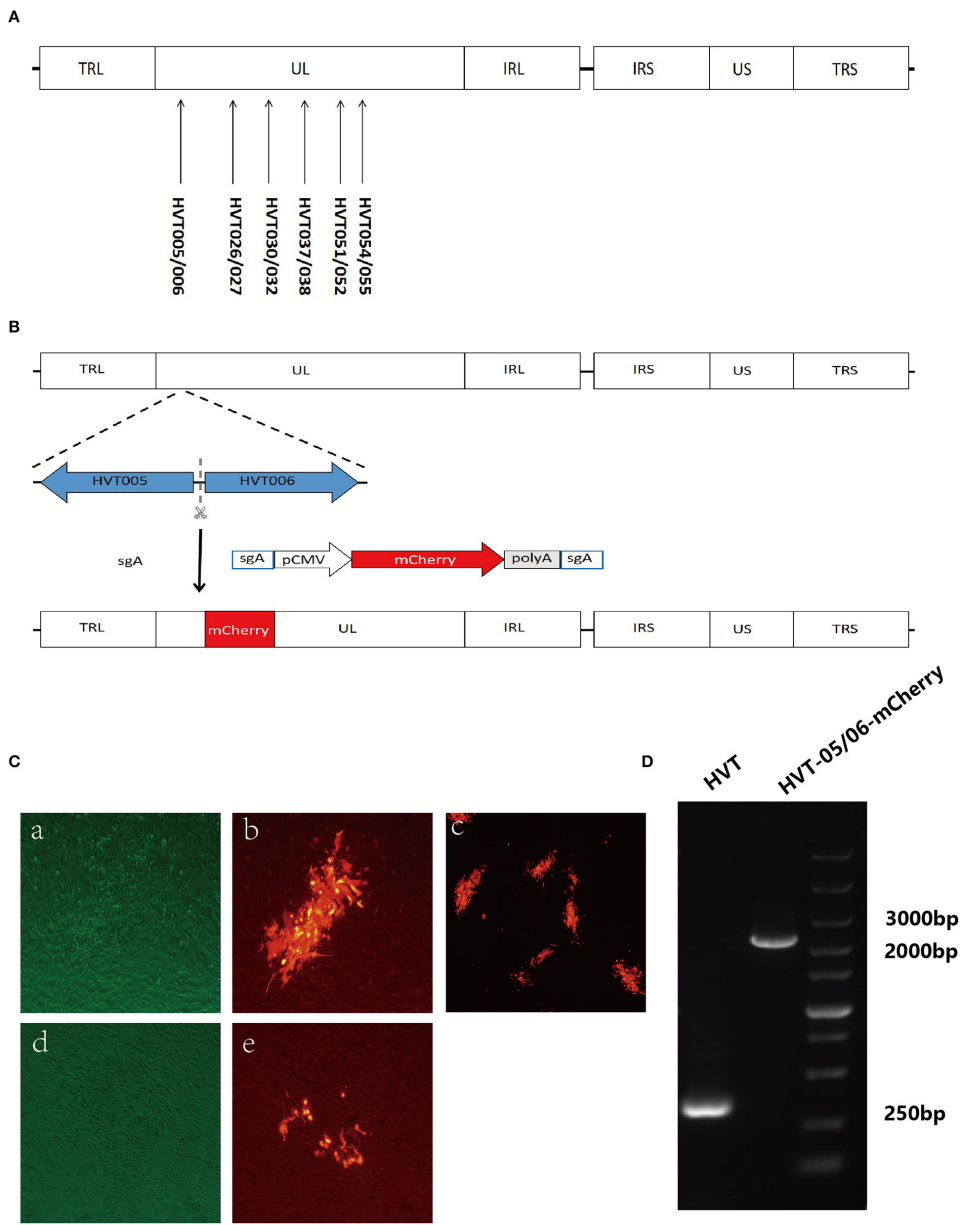
Screening for new insertion sites in the UL region of the HVT genome and construction of recombinant HVT-mCherry. **(A)** Location of the new insertion sites in the HVT genome. **(B)** Schematic presentation of the construction of recombinant HVT-005/006-mCherry. The mCherry cassette was flanked by sgA target sites and inserted into HVT-005/006 site. **(C)** Examples of stable and unstable HVT-mCherry at the first round of purification. CEF cells infected with HVT-005/006-mCherry (stable) under bright field with the green filter (a), fluorescence field merged with bright field (b), and fluorescence field (c). CEF cells infected with HVT026/027-mCherry (unstable) under bright field with the green filter (d) and fluorescence field merged with bright field (e). **(D)** PCR products of the mCherry cassette inserted in the HVT-005/006 site.

To identify new loci potentially valuable for the insertion of foreign genes, the list of all the intergenic sites in the UL region of the HVT genome (Genbank accession number: AF291866.1), except for the previously reported sites such as HVT053/054, was generated. Subsequently, all the sequences of these sites were subjected to the sgRNA design web (http://crispor.tefor.net/) for searching the suitable gRNAs. The sgRNAs were also checked using CHOPCHOP (http://chopchop.cbu.uib.no/) and CRISPOR (http://crispor.tefor.net/). Based on the scoring result of each search, 14 sgRNAs from six sites (Figure 1A) were selected for gene editing to develop recombinant HVT using methods described previously (Tang et al., 2018). The DNA oligo of sgRNAs (Table 1) was synthesized and cloned into the plasmid pX330A-1X2 (Addgene, Watertown, USA, #58766) containing the *BbsI* restriction site. The sgA sequence referred from the published data (He et al., 2016) was cloned into px459-v2 (Addgene, Watertown, USA, #118632) in a similar manner. The fragment sgA+mCherry cassette+sgA was amplified from plasmid pCMV-C-mCherry (Beyotime, Shanghai, China) using a primer pair of Doner-mCherry-F and Doner-mCherry-R (Table 2) and inserted into pGEM®-T Easy Vector (Promega, Beijing, China) to construct the plasmid pT-sgA-mCherry (containing the element of sgA+mCherry cassette+sgA). Plasmids pcDNA3.1(+)-SfiIx2 and pGEM-sgA-LoxP-GFP were constructed previously (Tang et al., 2018).

**Table 1:** sgRNA targeting sequences of the HVT genome and the donor plasmid.

**sgRNA**	**Target sequences**	**PAM**	**Locus**
HVT005/006-1	TCAGTATATGAAATTGATTG	TGG	Between HVT005 and HVT006
HVT005/006-2	TCATATACTGAATCGTAGGG	CGG	
HVT026/027-1	GAGAACTAATTATGGCACCG	TGG	Between HVT026 and HVT027
HVT030/032-1	GTGAAGGTCAACCTAACAGG	CGG	Between HVT030 and HVT032
HVT037/038-1	CGCATGTTTAAAACAGACCG	CGG	Between HVT037 and HVT038
HVT037/038-2	ACGTAAGCTAGTGTCACCAA	AGG	
HVT037/038-3	GGTGACACTAGCTTACGTGG	GGG	
HVT037/038-4	TGACACTAGCTTACGTGGAG	GGG	
HVT037/038-5	GTACCGCCACAACTACAATG	TGG	
HVT051/052-1	TTGAGCGGTCGAAAACAATG	AGG	Between HVT051 and HVT052
HVT054/055-1	TGGTCTTCGGTAGAGCAGGG	CGG	Between HVT054 and HVT055
HVT054/055-2	CGGTTGCTGATTAAGACGCG	CGG	
HVT054/055-3	GAAGCAATGTGGTTGATGGG	CGG	
HVT054/055-4	ATTCAGCCGCGGAAGCAATG	TGG	
sgA	GAGATCGAGTGCCGCATCAC	CGG	sg-A

**Table 2:** Primer sequences used for donor template construction.

**Primer**	**Sequences**
Donor-mCherry-F	GAGATCGAGTGCCGCATCACCGGTTTGCTGGCCTTTTGCTCAC
Donor-mCherry-R	GAGATCGAGTGCCGCATCACCGGGCCGATTTCGGCCTATTGGT
HA-NOT1-F	ATTTGCGGCCGC ATGGAGGCAGTATCACTAATAAC
HA-NOT1-R	ATTTGCGGCCGC TTATATACAAATGTTGCATCTGC

The donor plasmid pGEM-sgA-LoxP-GFP-HA, used for HA insertion, was constructed in several steps. First, the HA gene was amplified from H9N2 virus strain A/chicken/China/H1/2019(H9) (Wang et al., 2021) using gene-specific primers containing the *NotI* restriction enzyme site listed in Table 2. Then the HA sequence was inserted into pcDNA3.1(+)-SfiIx2 *via NotI* site to generate pcDNA3.1(+)-SfiIx2-HA. After that, the HA expression cassette released from pcDNA3.1(+)-SfiIx2-HA by *SfiI* restriction enzyme sites was transferred into pGEM-sgA-LoxP-GFP *via* two *SfiI* sites to generate pGEM-sgA-LoxP-GFP-HA.

### Generation of the Recombinant HVT-mCherry Virus

Primary CEFs were plated into 24-well plates the day before transfection. For one well, Cas9/gRNA expression plasmids targeting both the HVT genome (0.25 μg) and the donor plasmid pT-sgA-mCherry (0.25 μg) were co-transfected with 0.25 μg of donor plasmid into CEF cells by using TransIT-X2® according to the manufacturer's protocol (Mirus Bio, Madison, USA). At 12 h post-transfection, CEF cells were infected with 5,000 plaque-forming units (PFU) of HVT per well. At 48 h post-infection, the infected CEFs were harvested for plaque purification by using a fluorescent marker.

For the purification of the HVT-mCherry virus, the plaque with the red fluorescent marker was picked, and the viral particles were transfected into new CEF cells (a plaque into two wells of a six-well plate). The process (indicating one round of purification) was repeated until all the plaques showed the red fluorescent marker.

### Generation of the Recombinant HVT-005/006-HA

The process of transfection and infection to generate recombinant HVT-005/006-HA-GFP was similar to the generation of the recombinant HVT-mCherry except for the use of the donor plasmid, pGEM-sgA-LoxP-GFP-HA, and the GFP marker for plaque purification. For the excision of GFP using Cre recombinase, 2 μg of pcDNA3-Cre was transfected into CEFs in the 24-well plate. At 24 h post-transfection, the cells were infected with 5,000 PFU of HVT. Two days later, the infected CEFs were harvested for plaque purification by picking GFP-negative plaques.

For the purification of the HVT-005/006-HA virus, the GFP-negative plaque was picked and the viral particles were transfected into new CEF cells (a plaque into two wells of a six-well plate). The process (indicating one round of purification) was repeated until all the plaques showed no fluorescent marker.

### Characterization of the Recombinant HVT-005/006-mCherry and HVT-005/006-HA Viruses

Chick embryo fibroblasts were plated in six-well plates and then infected with HVT, HVT-005/006-mCherry, and HVT-005/006-HA the following day. The infected cells were harvested at 72 h post-infection and lysed in 1 × squishing buffer (10 mM Tris-HCl, pH 8, 1 mM EDTA, 25 mM NaCl, and 200 mg/ml Proteinase K) at 65°C for 30 min and 95°C for 2 min. PCR targeting the insertion region was carried out using a primer pair of T-005/006-F and T-005/006-R to identify the correct knock-in of the mCherry and HA expression cassettes in the recombinant HVT-005/006-mCherry and HVT-005/006-HA, respectively. The primers T-HA-F and T-HA-R were used for amplifying a part of the HA sequence. The sequence of primers is listed in Table 3.

**Table 3:** Primers used for knock-in detection.

**Primer**	**Sequences**
T-005/006-F	TCGTTTGCGCGTAGTAACATT
T-005/006-R	TAACTGTGAGCAATGCAGGGG
T-HA-F	CTATTCGGGGCCATAGCAGG
T-HA-R	GGTCCCGTTCCGAATTGTCT

### Western Blot Analysis

The expression of HA protein in the HVT-005/006-HA was detected by Western blot analysis. CEFs grown in six-well plates were infected with HVT and HVT-005/006-HA (10,000 PFU per well), respectively. On day 4 post-infection, the cells were harvested and lysed in 1% SDS using a protease inhibitor cocktail. Because the anti-HA(H9) monoclonal antibody (MAb) (clone 2G4 generated at Key Laboratory of Jiangsu Preventive Veterinary Medicine, Yangzhou University, China) (Wan et al., 2014) recognizes the conformation epitope, it has a better combination with the HA protein after non-reducing SDS-PAGE. The cell lysates were treated with the non-reducing SDS-PAGE loading buffer. After subjecting the samples to non-reducing SDS-PAGE, the separated proteins were transferred to nitrocellulose membranes. The membrane was blocked with 5% (w/v) skimmed milk dissolved in PBST containing 0.1% Tween-20 before being probed with anti-HA(H9) monoclonal antibody (MAb) and HRP-labeled goat anti-mouse antibody. The HA protein bands were visualized using the ECL detection reagent (Super Signal West Pico; Thermo Fisher Scientific, Shanghai, China) and the FluorChemE imaging system (Protein Simple, Wallingford, USA).

### Indirect Immunofluorescence Analysis (IFA)

The expression of HA in the recombinant virus was evaluated by immunofluorescence assays using fluorescence microscopy. CEF cells grown in 24-well plates were infected with HVT and the recombinant virus HVT-005/006-HA. After 72 h post-infection, virus-infected cells were fixed with 4% paraformaldehyde and treated with 0.25% Triton X-100. Anti-H9 chicken serum (generated at Key Laboratory of Jiangsu Preventive Veterinary Medicine, Yangzhou University, China) was used for detecting the HA expression, and goat anti-chicken IgY (H+L) labeled with Alexa Fluor 568 (Thermo Fisher Scientific, Shanghai, China) served as the secondary antibody. Anti-gB monoclonal antibody (MAb) (clone BD8) (Lu et al., 1997) was used for the detection of the gB expression, and goat anti-mouse IgG (H+L) labeled with Alexa Fluor 488 (Thermo Fisher Scientific, Shanghai, China) served as the secondary antibody.

### Stability of the Recombinant HVT-005/006-HA Virus

The recombinant virus HVT-005/006-HA was grown sequentially in CEF cells for 15 passages, and the integrity of the HA gene was examined by PCR using a DNA sample extracted from every fifth passage. The expression of the HA gene and HVT gB gene was detected by IFA at the 15th passage.

### Viral Growth Kinetics

To investigate the growth properties of the HVT-005/006-HA, CEFs in six-well plates were infected with 100 PFU per well of HVT and the recombinant HVT-005/006-HA. CEFs were harvested at 24, 48, 72, 96, and 120 h post-infection. To titrate the viruses, CEFs seeded in six-well plates were inoculated with 10-fold dilutions (10^−1^ to 10^−6^) of HVT and HVT-005/006-HA. Each dilution was done in duplicate wells containing CEFs. On day 4 post-infection, cells were checked by IFA, and the fluorescent plaques after staining were analyzed and counted using the inverted fluorescence microscope at the dilution that gave the easiest distinction between plaques. The titer of each sample was tested four times. The statistical difference in PFU between HVT and HVT-005/006-HA cells was compared and analyzed by unpaired *t*-tests.

## Results

### Screening of Insertion Sites for Generation of the Recombinant HVT-mCherry

To determine whether the sgRNA sites (Table 1) were suitable for the insertion of foreign genes, all the listed sgRNAs were used for the generation of recombinant HVT-mCherry. The workflow involved in the generation of recombinant HVT-mCherry is shown in Figure 1B, using the insertion of the HVT-005/006 site as an example. There were three possible outcomes for each sgRNA, that is, generating a recombinant virus with (1) stable expression, (2) unstable expression, or (3) no recombinant virus, each of which could be distinguished by fluorescent microscopy (Figure 1C). Only sgRNA HVT-005/006-2 generated recombinant HVT that stably expressed mCherry. Other sgRNAs (HVT026/027-1, HVT030/032-1, HVT037/038-5, HVT051/052-1, and HVT054/055-2) generated unstable HVT with mCherry expressing virus obtained only at the first round of the purification. Purified stocks of these recombinant viruses could not be obtained due to the instability of the mCherry expression in these insertion sites. Other sgRNAs did not generate any recombinant HVT-mCherry construct (Table 4). For the recombinant HVT-005/006-mCherry virus, fluorescent microscopy demonstrated red fluorescent plaques on CEF. PCR using primers outside of the HVT-005/006 insertion site gave positive results with the expected sized products (Figure 1D), confirming that the HVT-005/006-mCherry is a purified virus without contamination with the wild-type HVT.

**Table 4:** Screening results of the mCherry insertion sites in the UL region of HVT.

**sgRNA**	**Number of the fluorescent plaques obtained at the first round of purification**	**Stability of the fluorescent virus**
HVT005/006-1	0	
HVT005/006-2	9	Stable
HVT026/027-1	4	Unstable
HVT030/032-1	5	Unstable
HVT037/038-1	0	
HVT037/038-2	0	
HVT037/038-3	0	
HVT037/038-4	0	
HVT037/038-5	2	Unstable
HVT051/052-1	1	Unstable
HVT054/055-1	3	Unstable
HVT054/055-2	0	
HVT054/055-3	0	
HVT054/055-4	0	

### Generation of the Recombinant HVT-005/006-HA

**Figure 2:**
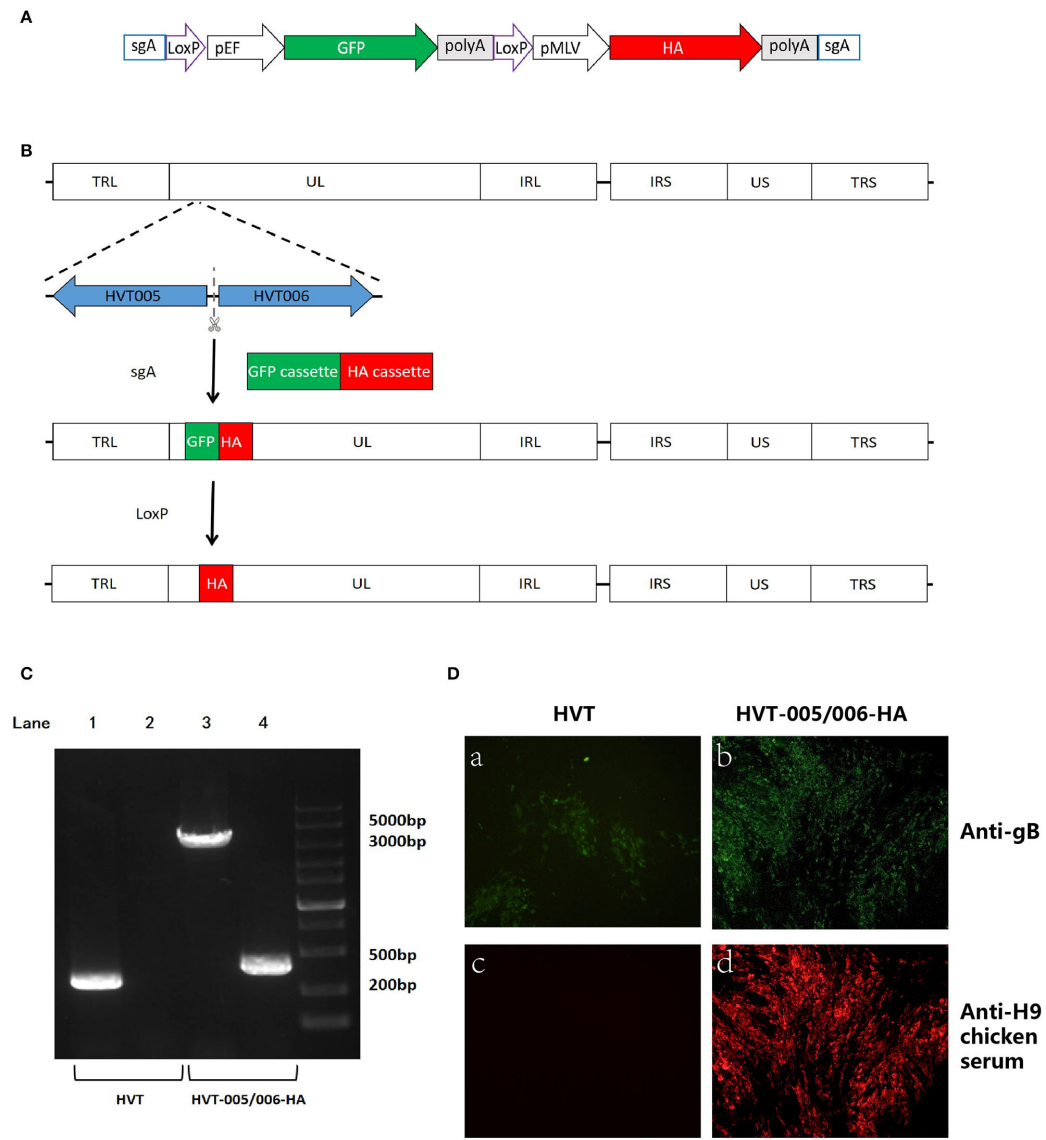
Construction and characterization of recombinant HVT-005/006-HA. **(A)** Schematic representation of the donor construct. The elements include Cas9 target site sgA, LoxP sequences, GFP cassette, and HA cassette. The sgA site was used for releasing the insertion fragment of the GFP cassette along with the HA cassette. The LoxP sequences were used for the excision of the GFP cassette. **(B)** Schematics of the strategy for insertion of HA cassette in the HVT-005/006 site. Both GFP and HA cassettes were inserted in the HVT-005/006 site to obtain the HVT-005/006-HA-GFP. HVT-005/006-HA was obtained after the removal of the GFP cassette by Cre recombinase. **(C)** PCR analysis of the inserted HA gene cassette using primer pairs outside the insertion HVT-005/006 site or amplifying part of the HA gene. Lane 1 PCR for the inserted HA cassette amplified from HVT DNA with the primer pair T-005/006-F and T-005/006-R, Lane 2 PCR for the part of HA sequence amplified from HVT DNA with the primer pair of T-HA-F and T-HA-R, Lane 3 PCR for the inserted HA cassette amplified from HVT-005/006-HA DNA with the primer pair T-005/006-F and T-005/006-R, and Lane 4 PCR for the part of HA sequence amplified from HVT-005/006-HA DNA with the primer pair of T-HA-F and T-HA-R. **(D)** Detection of HA expression with IFA using anti-H9 chicken serum. HVT infection was confirmed by immunostaining with anti-gB monoclonal antibody BD8. IFA for CEFs infected with HVT using anti-gB monoclonal antibody BD8 **(a)**. IFA for CEFs infected with HVT-005/006-HA using anti-gB monoclonal antibody BD8 **(b)**. IFA for CEFs infected with HVT using anti-H9 chicken serum **(c)**. IFA for CEFs infected with HVT-005/006-HA using anti-H9 chicken serum **(d)**.

For the generation of recombinant HVT-005/006-HA, an AIV H9HA expression cassette was inserted into the HVT-005/006 of the HVT genome to generate the recombinant virus. The strategy for insertion of the HA expression cassette was carried out as described previously (Tang et al., 2018). Briefly, as shown in Figures 2A,B, the recombinant HVT was constructed by including a fluorescent marker GFP, which was later removed by the Cre recombinase, along with the HA expression cassette. For the recombinant HVT, correct insertion and purity of the purified virus were examined by PCR with one pair of primers located outside of the insertion site and another pair amplifying the part of the HA sequence. As shown in Figure 2C, a 465-bp HA band amplified from HVT-005/006-HA DNA was clearly seen, while no HA band was detected in the control lane loaded with the wild-type HVT DNA sample. As for the purity of the recombinant HVT, only the HA cassette band (about 3,500 bp) was obtained with no wild-type virus DNA band present.

### Characterization of the HVT-005/006-HA

**Figure 3:**
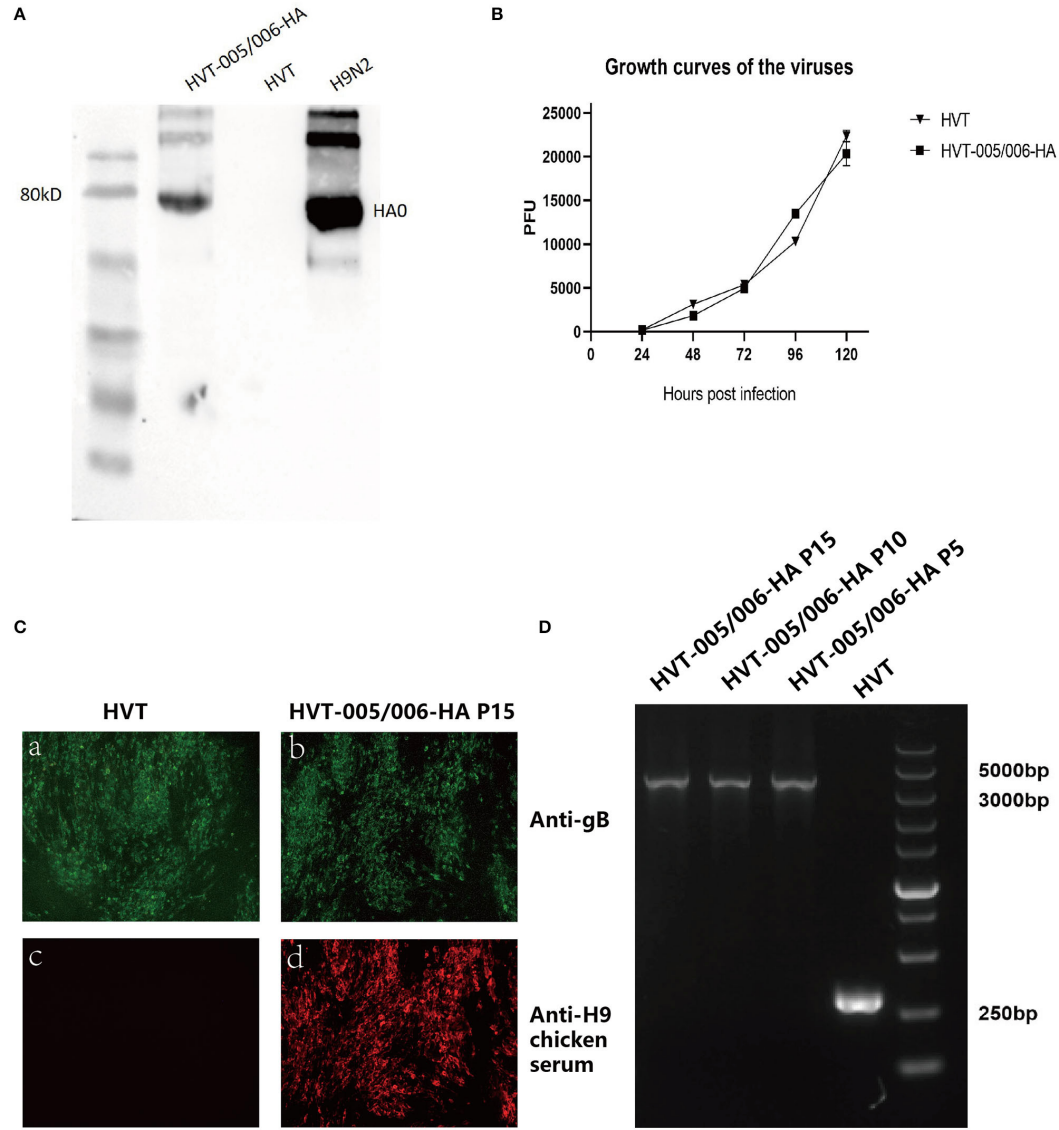
Characterization of recombinant HVT-005/006-HA. **(A)** Detection of HA expression by Western blot. **(B)** Growth curves of HVT-005/006-HA and HVT. HVT-005/006-HA and HVT viruses were inoculated into CEFs seeded in 12-well plates with 100 PFU per well. Cells were harvested and titrated at 24, 48, 72, 96, and 120 h post-infection. **(C)** Detection of HA expression from HVT-005/006-HA at passage 15 in CEFs by IFA using anti-H9 chicken serum. HVT infection was confirmed by immunostaining with anti-gB monoclonal antibody BD8. IFA for CEFs infected with HVT using anti-gB monoclonal antibody BD8 **(a)**. IFA for CEFs infected with HVT-005/006-HA P15 using anti-gB monoclonal antibody BD8 **(b)**. IFA for CEFs infected with HVT using anti-H9 chicken serum **(c)**. IFA for CEFs infected with HVT-005/006-HA P15 using anti-H9 chicken serum **(d)**. **(D)** PCR analysis to confirm the presence of HA expression cassette from HVT-005/006-HA at passages 5, 10, and 15 in CEFs using primer pair outside of the insertion site.

To investigate whether the inserted HA gene could be expressed in the CEFs infected with recombinant HVT-005/006-HA, IFA and Western blot analyses were carried out using the HA-specific antibody. For IFA, the HA was detected in recombinant virus-infected cells in contrast to cells infected with wild-type HVT (Figure 2D). Western blot analysis showed the presence of an HA0 band (~75 kD) in the HVT-005/006-HA and H9N2 allantoic fluid samples, while no HA0 band was detected in the HVT-infected cell lysate (Figure 3A). Both IFA and Western blot analysis demonstrated the expression of HA protein specifically in the CEFs infected with HVT-005/006-HA compared with the wild-type HVT.

The growth kinetics of HVT-005/006-HA was also measured to determine whether the HA insertion into the HVT-005/006 locus affected the replication ability of the recombinant virus *in vitro*. Statistically, data between HVT and HVT-005/006-HA did not have a significant difference (*P* > 0.05). Meanwhile, as demonstrated in Figure 3B, the growth curve of HVT-005/006-HA was similar to that of wild-type HVT, indicating that the recombinant HVT grew at a similar level as the parental HVT in CEFs.

### Stability of the Recombinant HVT-005/006-HA Virus

The genetic stability of the HA expression cassette was measured by passing HVT-005/006-HA sequentially in CEF cells for 15 passages. After every five passages, the expression of the HA gene was determined by IFA, and the viral DNA was extracted and analyzed by PCR. For IFA, the HA was detected in the recombinant virus-infected cells in contrast to the cells infected with wild-type HVT (Figures 3Cc,Cd), while the gB was detected in both HVT and HVT-005/006-HA virus-infected cells (Figures 3Ca,Cb). PCR results (Figure 3D) also showed that only the HA cassette was amplified from the 5th, 10th, and 15th passage of the HVT-005/006-HA DNA samples, while no wild-type PCR band appeared. IFA and PCR results indicated the stable integration of the HA gene in the HVT-005/006 locus of the HVT genome even after 15 passages.

## Discussion

There are two pathways, including homology-directed repair (HDR) and non-homologous end joining (NHEJ), for the generation of recombinant HVT vaccines by CRISPR/Cas9 system. In non-homologous DNA end joining (NHEJ), the DSB is first recognized by the Ku70–Ku80 heterodimer (Ku). Then the Ku70–Ku80 heterodimer recruits other NHEJ proteins, such as NHEJ polymerase, nuclease, and ligase complexes, to promote the joining of DNA ends. With the guide of microhomology between the two DNA ends and the action of resecting and adding nucleotides done by these enzymes, the DSB of the DNA is repaired. The process is error-prone and can result in diverse DNA sequences at the repair junction (Chang et al., 2017). For homology-directed repair (HDR), the MRN–CtIP complex resects on the break to generate single strands at the 3' ends of the DNA molecule. After resection, the NHEJ is inhibited. The ssDNA is first combined by RPA, which is replaced by Rad51 later. Rad51 forms a nucleoprotein filament on single-stranded DNA after end resection, which mediates strand invasion on the homologous template. With the extension of the D-loop and capture of the second end, the DSB is repaired (Jasin and Rothstein, 2013). HDR typically occurs at lower frequencies than NHEJ because it is generally active only in dividing cells, while NHEJ occurs throughout the cell cycle (Saleh-Gohari and Helleday, 2004; Chang et al., 2017). In this study, NHEJ-dependent CRISPR/Cas9 editing was used to screen the insertion sites and generate HVT recombinants for two reasons. One is that though HDR-dependent CRISPR/Cas9 provides precise genome editing, when considering the insertion of the expression cassette, precise insertion is a less important factor when compared to the high efficiency of NHEJ. Meanwhile, the study involved the insertion of the genome at several sites, and NHEJ-dependent CRISPR/Cas9 editing is easier and more convenient to generate HVT recombinants, as it enables the repeated use of the donor plasmid.

The method used in this study to screen the HVT genome for new insertion sites is rapid and convenient, as it took only about 2 weeks to screen for suitable insertion sites in the HVT genome: 3 days to make the virus stock (collecting the infected cells after transfection and infection) and 12 days to confirm whether the recombinant virus clone is stable (3–4 rounds of purification). In this study, all the sgRNAs targeting six new intergenic sites were used to insert the mCherry cassette. Only the HVT-005/006 locus was proven to be a stable site for the insertion, as most of the other loci could not sustain stable expression of mCherry when the virus was passaged. Some loci also failed to produce any recombinant viruses. In the current study, we have only explored the intergenic loci in the UL region of the HVT genome for potential sites of insertion. This method could be applied to screen the insertion sites of the whole HVT genome, as well as the genomes of other avian herpesvirus vaccine strains, such as CVI988, which is another promising vector platform for generating recombinant viruses.

As a viral vector, HVT is widely used for expressing different immunogenic antigens of poultry pathogens, such as NDV, IBDV, and AIV. Apart from bivalent vaccines based on HVT vector, double insert HVT-Vectored Vaccines such as Innovax-ND-IBD (Merck Animal Health), Vaxxitek HVT+IBD+ND, and Vaxxitek HVT+IBD+ILT (Boehringer Ingelheim) were licensed for use in poultry (Francis, 2021). However, the development of multivalent recombinant HVT vaccines is limited by the availability of suitable insertion sites that are also restricted by commercial intellectual property (IP) restrictions. Identification of the HVT-005/006 locus as a potential insertion site for the stable expression of foreign genes in this study will help to advance the drive for generating recombinant HVT viruses (Zhang et al., 2014). The 159-kb double-stranded DNA of the HVT genome contains several non-essential regions for viral replication. By replacing these non-essential genes with genes from other pathogens, HVT can be developed further as a potent viral vector platform for the development of polyvalent live vaccines against poultry diseases. Apart from these non-essential genes, such as US2, US10, and UL2 (Tsukamoto et al., 2002; Gao et al., 2011), the intergenic region of the HVT genome was also considered as the site for the insertion of heterologous antigen genes, such as UL45/UL46 and HVT065/HVT066, which have also been proven to be suitable for the foreign gene insertion (Tang et al., 2020). With the widespread prevalence of avian pathogens, such as H9N2 AIV, and the shortcomings of inactivated vaccines, there is an urgent demand for developing new vaccines to control the circulation of H9N2 AIVs in many parts of the world, including China. A commercial vaccine, the AIV H5HA vaccine (Vectormune® AI, CEVA Animal Health, Lenexa, KS, USA), based on the HVT vector has been demonstrated to provide clinical protection and significantly reduce the shedding of challenge virus in the vaccinated chickens when challenged with heterologous highly pathogenic H5 AIV (Palya et al., 2018). Since then, several recombinant HVT-vectored vaccines expressing HA of AIV have been generated by researchers. Many of them focused on the UL45/46 region of the HVT genome, which has been proven to be a good site for inserting the HA gene by this commercial AIV H5HA vaccine. Unlike this commercial AIV vaccine for H5HA, researchers tried to insert the HA(H7) or HA(H9) into this site. Apart from UL45/46 region, other sites that had been reported were US2 and US10 regions, but US2 was better than US10 (Gao et al., 2011; Li et al., 2011; Chang et al., 2019; Liu et al., 2019; Tang et al., 2020). Most recombinant HVT-vectored vaccines showed effective protection in vaccinated chickens when challenged with homologous AIV. It is known that protection can be correlated with HI serological titers, and the HI serological titer results in these studies demonstrated that antigen-specific antibodies could be induced by these recombinant HVT viruses (Gao et al., 2011; Li et al., 2011; Kapczynski et al., 2015, 2016; Hamad et al., 2018; Liu et al., 2019). Apart from this, the recombinant HVT virus could induce cellular immunity, which is also involved in the protection of chickens. By the examination of T lymphocytes expressing IFN-γ in the lungs, Liu et al. (2019) found that rHVT-H9 could induce robust cell immune responses compared to HVT (Liu et al., 2019). The study by Kapczynski et al. (2015) also confirmed this point. The rHVT-Hu4999 could also provide good protection when vaccinated chickens were challenged with heterologous viruses, though the HI titers were much lower, with the average between 2^1^ and 2^3^ using heterologous HA antigens (Kapczynski et al., 2015). Overall, the recombinant HVT-vectored vaccine may be an ideal live vaccine candidate to control the H9N2 infection, and we would evaluate the efficacy of HVT-005/006-HA in further study.

In this study, we identified a new insertion site (HVT-005/006) and successfully constructed the HVT-005/006-HA vector. Indirect immunofluorescence analysis and Western blot analysis showed that HA gene exhibited good expression in the HVT-005/006 site. The PCR results confirmed that the HA cassette integrated into the HVT-005/006 location remained stable even after 15 passages. Meanwhile, HVT-005/006-HA showed similar growth to the wild-type virus, confirming the insertion of the HA expression cassette did not have any effect on HVT replication. All the results demonstrated the suitability of this site for the insertion of the HA gene, and HVT-005/006-HA may be a good candidate for the prevention and control of H9N2 influenza in the future. Although the insertion of the foreign antigen of HA was tested here, it will be feasible to use the HVT-005/006 as an insertion site to express other foreign antigens, which has a significant implication in the construction of bivalent and even multivalent recombinant vaccines.

To our knowledge, identification of the HVT-005/006 locus as a potential insertion site for stable expression of foreign genes in a recombinant HVT has not been reported previously. While we have shown the use of this locus for stable expression of the AIV HA gene, it can be potentially applied to express antigens derived from other important avian pathogens, such as the fusion (F) or hemagglutinin-neuraminidase (HN) of NDV, glycoprotein D (gD) and glycoprotein I (gI) of ILTV, and viral protein 2 (VP2) of IBDV. Furthermore, this insertion site can be used with other reported insertion sites, such as UL45/46 or/and US2, to construct multivalent recombinant vaccines.

## Data Availability Statement

The original contributions presented in the study are included in the article/supplementary material, further inquiries can be directed to the corresponding author/s.

## Author Contributions

This manuscript was written by XZ and AQ. Experiment and data analysis were performed by XZ and BS. The study was designed by XZ, AQ, JY, KQ, HS, YY, and VN. All authors contributed to the article and approved the submitted version.

## Funding

This project was supported by the National Science Foundation of China (31972717), Yangzhou University International Academic Exchange Fund (grant YZUIAEF201802020), the Biotechnology and Biological Sciences Research Council (BBSRC; grants BB/R012865/1 and BBS/OS/NW/000007), the Priority Academic Program Development of Jiangsu Higher Education Institutions, and the Jiangsu Co-innovation Center for the Prevention and Control of Important Animal Infectious Diseases and Zoonoses. The funding bodies did not play direct roles in the design of the study and collection, analysis, and interpretation of the data and writing of the manuscript.

## Conflict of Interest

The authors declare that the research was conducted in the absence of any commercial or financial relationships that could be construed as a potential conflict of interest.

## Publisher's Note

All claims expressed in this article are solely those of the authors and do not necessarily represent those of their affiliated organizations, or those of the publisher, the editors and the reviewers. Any product that may be evaluated in this article, or claim that may be made by its manufacturer, is not guaranteed or endorsed by the publisher.
